# Acupoint injection in improving pain and joint function of knee osteoarthritis patients: A protocol for systematic review and meta-analysis

**DOI:** 10.1097/MD.0000000000024997

**Published:** 2021-03-26

**Authors:** Houyi Wang, Jinxu Sun, Xiuying Yu, Yong He

**Affiliations:** Chinese Medicine Hospital in Linyi City, Linyi, Shandong Province, China.

**Keywords:** acupoint injection, knee osteoarthritis, meta-analysis, protocol, systematic review

## Abstract

**Background:**

Knee osteoarthritis is a common chronic progressive disease, which seriously affects the quality of life of the middle-aged and elderly, and even leads to disability. More and more evidence shows that acupoint injection is beneficial to the clinical treatment of knee osteoarthritis, but there are differences in the efficacy of different acupoints and injection drugs, and there is no systematic review to assess this therapy at present. The purpose of this study is to systematically evaluate the efficacy and safety of acupoint injection in improving pain and joint function in patients with knee osteoathrosis.

**Methods:**

According to the retrieval strategy, we will search from CNKI, Wanfang, VIP, Chinese Biomedical Science, PubMed, Embase, Web of Science and the Cochrane Library for randomized controlled trials of acupoint injection in the treatment of knee osteoarthritis from the establishment of the database to February 2021. The study will be screened according to the inclusion and exclusion criteria, and the Cochrane risk bias assessment tool will be used to evaluate the quality of the study. Revman 5.4 software is used for meta-analysis.

**Results:**

This study will evaluate the efficacy of acupoint injection in the treatment of knee osteoarthritis by evaluating the total effective rate, the degree of pain relief, joint function score, adverse reactions, and so on.

**Conclusion:**

This study will provide reliable evidence-based basis for the clinical application of acupoint injection in the treatment of knee osteoarthritis.

**Ethics and dissemination:**

Private information from individuals will not be published. This systematic review also does not involve endangering participant rights. Ethical approval will not be required. The results may be published in a peer-reviewed journal or disseminated at relevant conferences.

OSF Registration number—doi: 10.17605/OSF.IO/M5FTK.

## 1 Introduction

Knee osteoarthritis is a common chronic osteoarthrosis in the middle-aged and elderly, which often causes joint pain, limitation of activity and decline in quality of life,^[1]^ which is the main cause of lower-limb disability in the elderly.^[2]^ Clinically, clinical symptoms of KOA include arthralgia, stiffness, movement limitation, occasional articular effusion, and varying degrees of local inflammation.^[3]^ Its etiology and pathogenesis have not been fully clarified, in addition to sex, age, occupation, heredity, but also related to excessive exercise and other factors.^[4]^ The main pathological features are articular cartilage defects and subchondral osteosclerosis caused by the synergistic action of mechanical and biological mechanisms.

At present, the standard treatment is mainly through analgesics and nonsteroidal anti-inflammatory drugs (NSAIDs) to alleviate symptoms, which can relieve pain and inhibit inflammatory transmitter reaction to a certain extent, but these drugs cannot effectively prevent the degeneration of knee cartilage, and long-term use may cause damage to the digestive system, liver and kidney function of the human body.^[5,6]^ Pain persists after drug therapy and other non-invasive interventions, and intra-articular injection can also be performed before surgical intervention. Glucocorticoid is the most commonly used intra-articular injection drug, which can reduce pain and improve function.^[7]^ However, local injection of glucocorticoid can cause a series of complications, which may limit the long-term use in patients, such as osteoporosis, local skin pigmentation, and so on.^[8,9]^ Therefore, it is necessary to find new complementary and alternative therapies to treat KOA.

Acupoint injection is an alternative supplementary therapy, it is based on the Chinese meridian theory, the drug will be injected into specific acupoints, through the stimulation of acupoints to achieve the purpose of treatment.^[10]^ Acupoint injection and meridian acupoint stimulation have a synergistic effect, and this method has better curative effect than traditional acupuncture or intra-articular injection.^[11]^ At present, it has been used in myofascitis, knee osteoarthritis, external humeral epicondylitis and other muscle and joint diseases.^[12–14]^ Its positive effect has attracted more and more public attention. Although a number of randomized controlled studies have confirmed that acupoint injection can effectively relieve pain and improve joint function in patients with knee osteoarthritis.^[15–19]^ However, due to the lack of critically assessed clinical evidence, limits the promotion of the program. Many patients still do not benefit from the treatment. Therefore, this study plans to systematically evaluate the efficacy and safety of acupoint injection in improving pain and joint function in patients with knee osteoarthritis.

## 2 Methods

### 2.1 Protocol register

This protocol of systematic review and meta-analysis has been drafted under the guidance of the preferred reporting items for systematic reviews and meta-analyses protocols (PRISMA-P). Moreover, it has been registered on open science framework (OSF) (Registration number—doi: 10.17605/OSF.IO/M5FTK).

### 2.2 Ethics

Since the programme does not require the recruitment of patients and the collection of personal information, it does not require the approval of the Ethics Committee.

### 2.3 Eligibility criteria

#### 2.3.1 Inclusion criteria

(1) For patients diagnosed as knee osteoarthritis who have not received surgical treatment, the diagnostic standard refers to the KOA diagnostic criteria established by the American Rheumatic Association or the Orthopaedic Association of Chinese Medical Association,^[20]^ gender, race, age, and occupation are not restricted.
(2) The treatment group is treated with acupoint injection (acupoints and injection drugs are not restricted), while the control group can be treated with other forms other than surgical treatment.
(3) The type of study is randomized controlled study, and the language is limited to Chinese and English.

#### 2.3.2 Exclusion criteria

(1) Repeatedly published papers.
(2) The literature is abstract, animal research, and cadaver study.
(3) Non-primary knee osteoarthritis: secondary KOA, such as rheumatism, trauma, etc.
(4) The relevant data cannot be extracted from the published results, and the original data cannot be obtained after contacting the author.
(5) The study of the discrepancy between the intervention measures and the research.

### 2.4 Outcome index

(1) Primary outcome measures: total effective rate, pain score (such as VAS, etc.).
(2) Secondary outcome measures: joint function score (such as Lysholm score, WOMAC score system, etc) and adverse reactions.

### 2.5 Retrieval strategy

The database is independently searched by two researchers from the establishment of the database in February 2021. The retrieval database includes CNKI, Wanfang, VIP, China Biomedical Database, PubMed, Embase, Web of Science and the Cochrane Library. Chinese keywords: “knee arthritis”, “degenerative knee arthritis”, “acupoint injection”, “water acupuncture”; English Keywords: “knee osteoarthritis”; “Senile osteoarthritis”; “Acupoint Injection”; “Acupuncture Point Injection” and so on. Included literature is independently selected by two researchers according to the inclusion and exclusion criteria; and if there are differences of opinion; they will be decided after consultation with the third researcher. The PubMed retrieval strategy is shown in Table 1.

**Table 1:** Search strategy in PubMed database.

Number	Search terms
#1	Acupoint Injection [Title/Abstract]
#2	Acupuncture Point Injection [Title/Abstract]
#3	Water acupuncture [Title/Abstract]
#4	#1 OR #2 OR#3
#5	knee osteoarthritis [MeSH]
#6	Knee Osteoarthritides [Title/Abstract]
#7	Osteoarthritis of Knee [Title/Abstract]
#8	Senile osteoarthritis [Title/Abstract]
#9	knee arthritis [Title/Abstract]
#10	#5 OR #6 OR #7 OR #8 OR #9
#11	#4 AND #10

### 2.6 Data screening and extraction.

**Figure 1:**
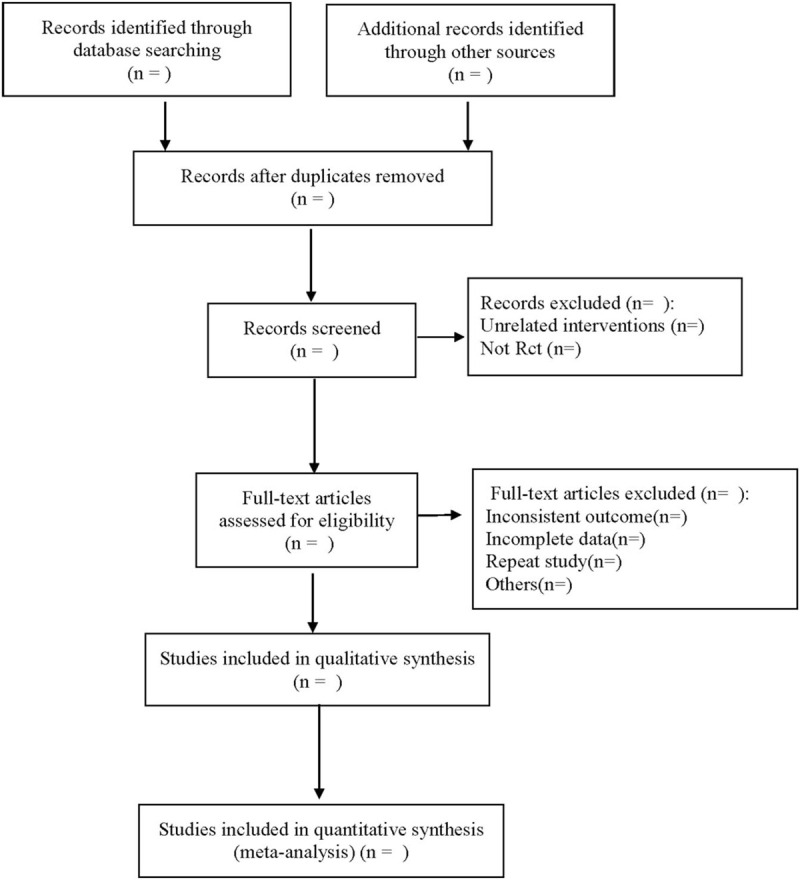
Flow diagram.

According to the above inclusion and exclusion criteria, two researchers read the title abstract to preliminaries screen out the literature that may meet the criteria, remove the repetitive literature, further read the full text, evaluate the quality of the literature independently, and finally determine the included literature. If there is a disagreement, the third researcher will discuss and decide together. The data include author, year of publication, type of study/level of evidence, a number of cases, stage of the disease, average age, follow-up time, country of the study, outcome index, and so on. The literature screening process is shown in Figure 1.

### 2.7 Literature quality assessment

The risk bias assessment tool of Cochrane collaboration Network is used to assess the risk bias of the included randomized controlled trials, including the following seven aspects: random sequence generation, allocation concealment, participant blinding, outcome assessor blinding, incomplete outcome data, selective reporting, and other sources of bias. Each domain was rated as having a high, low, or unclear risk of bias as appropriate

### 2.8 Statistical analysis

#### 2.8.1 Data analysis and processing

RevMan 5. 3 software is used for meta-analysis. The heterogeneity of each literature is judged by *x*^2^ test and *I*^2^, and the heterogeneity of the effect value is analyzed. If *P* *>* .1*, I*^*2*^ *<* 50%, it shows that the heterogeneity among the studies is low, so a fixed model is used for analysis; if *P* *<* .1*, I*^*2*^* ≥* 50%, there is obvious heterogeneity among the studies. The sources of heterogeneity are analyzed and the random effect model is used for analysis. The measurement data are expressed as the weighted mean difference (WMD) or the standard mean difference (SMD) and 95%CI, and the counting data are expressed as relative ratio (RR) and 95%CI.

#### 2.8.2 Dealing with missing data

If the data required for the study are incomplete or not reported in the study, the researcher will contact the lead author or newsletter author of the study by phone or email. If the required data are not available, we will use descriptive analysis instead of meta-analysis and exclude these studies if necessary.

#### 2.8.3 Subgroup analysis and sensitivity analysis

When the data permit, we will conduct subgroup analysis according to the follow-up time, disease stage, type of injection, and other factors; in order to test the stability of the meta-analysis results of each index, we will use one by one elimination method for sensitivity analysis.

#### 2.8.4 Assessment of reporting biases

In the meta-analysis, when data from at least 10 studies are available, we will consider generating a funnel chart to assess publication bias.

#### 2.8.5 Evidence quality evaluation

We will use the grading of recommendation assessment, development and evaluation (GRADE) scoring method to grade the evidence of the outcome index.^[21]^ Based on randomized controlled trials, the “high” quality rating is the highest level of evidence. The evaluation content includes bias risk, indirectness, inconsistency, inaccuracy, and publication bias, and the quality of evidence will be rated as high, medium, low or very low.

## 3 Discussion

Knee osteoarthritis is a chronic progressive disease that affects 20% of people over the age of 45.^[22]^ The current conservative treatment regimens have short-term effects, and each has its own advantages and disadvantages.^[23,24]^ However, the recurrence of chronic pain in knee OA and progressive joint dyskinesia prompt us to evaluate new alternative treatments.

Acupoint injection is a new acupuncture therapy, which combines acupuncture and drug therapy, so that drugs are directly applied to acupoints, which not only plays the role of medicine but also plays the role of meridian conduction, and regulates the meridian system of the human body through drug stimulation. Therefore, a better effect can be achieved. Drugs commonly used for acupoint injection include herbal extracts, vitamins, bee venom, placental extracts, etc.^[11,12,25]^ Modern studies have confirmed that common injection drugs, such as Weilingxian injection, can inhibit the overexpression of matrix metalloproteinases, reduce the dissolution of matrix metalloproteinases on joint tissue cells, and increase the secretion of hyaluronic acid. To lubricate the joint and promote inflammatory absorption.^[26]^ Salvia miltiorrhiza extract has many effects, such as dilating blood vessels, increasing tissue blood flow, improving microcirculation, antibacterial, immune regulation, a hormone-like, antioxidation, and so on.^[27]^ At present, there are many clinical studies on acupoint injection in the treatment of knee OA, and positive results have been obtained, but there are differences in the efficacy of different acupoints and injecting drugs, and there is no systematic and comprehensive meta-analysis on this topic. Through this systematic evaluation, we will provide objective evidence-based basis for the efficacy and safety of acupoint injection in the treatment of knee OA.

However, there are some limitations in our research: due to the limitations of language retrieval, we will only include Chinese and English literature, and may ignore studies in other languages and regions; different arthritis stages, acupoints, injecting drugs and other factors will increase the possibility of heterogeneity.

## Author contributions

**Data curation:** Houyi Wang, Jinxu Sun.

**Funding acquisition:** Yong He.

**Resources:** Houyi Wang, Xiuying Yu.

**Software:** Xiuying Yu.

**Supervision:** Jinxu Sun.

**Writing – original draft:** Houyi Wang, Jinxu Sun.

**Writing – review & editing:** Houyi Wang, Yong He.
